# Risky Food Safety Behaviors Are Associated with Higher Bmi and Lower Healthy Eating Self-Efficacy and Intentions among African American Churchgoers in Baltimore

**DOI:** 10.1371/journal.pone.0052122

**Published:** 2012-12-20

**Authors:** Elizabeth Anderson Steeves, Ellen Silbergeld, Amber Summers, Lenis Chen, Joel Gittelsohn

**Affiliations:** 1 Center for Human Nutrition, Johns Hopkins Bloomberg School of Public Health, Baltimore, Maryland, United States of America; 2 Department of Environmental Health Sciences, Johns Hopkins Bloomberg School of Public Health, Baltimore, Maryland, United States of America; 3 Department of Health, Behavior and Society, Johns Hopkins Bloomberg School of Public Health, Baltimore, Maryland, United States of America; University of Missouri-Kansas City, United States of America

## Abstract

**Background:**

There are an estimated 9.4 million cases of foodborne illness each year. Consumers have a key role in preventing foodborne illness, but differences in the practice of food safety behaviors exist, increasing risk for certain groups in the population. Identifying groups who are more likely to practice risky food safety behaviors can assist in development of interventions to reduce the disease burden of foodborne illnesses. The purpose of this investigation was to examine the relationships of health indicators and psychosocial factors with self-reported food safety behaviors.

**Methods and Findings:**

Data were collected via questionnaire from 153 African Americans who attend churches in Baltimore City. Individuals reported high overall concern with food safety (mean score: 0.80±0.49 on a scale of −1 to +1) and practiced food safety behaviors with moderate overall frequency (mean score: 5.26±4.01 on a scale of −12 to +12), with considerable variation in reported frequencies depending on the food safety behavior. After adjusting for demographic variables, food safety behaviors were significantly associated with BMI and psychosocial variables. Riskier food safety behaviors were associated with higher body mass index (BMI) (β = −0.141 95%CI (−0.237, −0.044), p = 0.004). Self-efficacy for healthy eating (standard β [std. β] = 0.250, p = 0.005) and healthy eating intentions (std. β = 0.178, p = 0.041) were associated with better food safety behaviors scores.

**Conclusions:**

These results show important relationships between weight-related health indicators, psychosocial factors and food safety behaviors that have not previously been studied. Interventions tailored to higher-risk populations have the potential to reduce the burden of food-related illnesses. Additional studies are needed to further investigate these relationships with larger and more diverse samples.

## Introduction

There are an estimated 9.4 million cases of foodborne illness that contribute to approximately 55,961 hospitalizations and 1,351 deaths each year [1]. While foodborne illnesses do not always cause a lasting impact, they can affect productivity, wellbeing, and health care expenditures, and in some cases they have the potential to create chronic, lifelong health problems such as kidney disease, arthritis, and digestive disorders [2]. Health care costs associated with illnesses caused by the most common foodborne bacteria equal $2.9 to $6.7 billion annually [3].

Regulations promulgated by both the United Stated Department of Agriculture (USDA) and Food and Drug Administration (FDA) exist to reduce risks of pathogen exposures of foods prior to reaching consumers; however, consumers are the last to handle food before consumption, and therefore have the final opportunity to decrease risk of foodborne illness by implementing proper food safety and consumption behaviors [4].

Consumers are often inconsistent in their practice of food safety behaviors. Studies using self-reported measures of food safety behaviors found that 21.5% of respondents report not consistently washing their hands before preparing food, 8.4% report not washing cutting surfaces after contact with raw meat or chicken [4], 11.9% practice improper refrigeration of leftovers, and 63% do not follow the guidelines for cooking foods to proper temperatures [5]. Studies using direct observation methods show that actual practices of food safety behaviors may be much worse than what is reported [6], [7]. A study observing individuals’ in-home food preparation found that 2/3 of the sample used inadequate hand washing and surface cleaning methods and almost all had some cross-contamination of foods [6].

The 2010 Dietary Guidelines for Americans contain a priority area emphasizing the need for improved food safety habits [8], and Healthy People 2020 has an objective for increasing the “proportion of consumers who follow key food safety practices” with sub-goals focused on washing hands and surfaces often, avoiding cross-contamination, cooking to proper temperatures, and refrigerating foods promptly [9].

Analyses of demographic variables related to consumer food safety behaviors and food consumption practices have been conducted to help identify those at highest risk of having poor food safety behaviors [10] and to develop targeted food safety messages for reducing risk in these groups. Previous research has explored associations of demographic variables such as gender[4], [10]–[12], age[4], [10]–[12], education level [4], [10], [13], [14], income [11], race/ethnicity [4], [10], metropolitan status [10], [11], and geographic location [10], [11] with food safety behaviors. Additional investigations have been conducted to determine relationships with variables of interest such as immune status [12] and role in meal preparation [4]. These studies have shown that men [4], [10]–[13] and individuals with higher incomes [11] and educational levels [4], [10]–[11], [13]–[14] tend to practice riskier food safety behaviors and food consumption practices than those who are female, less educated, and have lower incomes [4], [10]–[14]. African Americans tend to practice food safety behaviors and consumption patterns that are equally as safe and highly comparable to other racial/ethnic groups [4], [10]. Mixed results have been found among age strata [4], [10], [12], with some evidence showing that younger and older individuals have riskier behaviors [4]. While these relationships are fairly consistently documented (for all variables except age), minimal effort has gone into expanding the understanding of relationships of food safety behaviors with other health-related variables, leaving significant gaps in the literature and room for improvement in targeted food safety campaigns.

It is plausible that food safety behaviors by their very nature are linked to health practices and to eating patterns. Implementing proper food safety behaviors requires increased awareness and attention to the food preparation process, knowledge of safe practices, and self-efficacy for being able to implement these behaviors. Examining specific relationships between food safety behaviors, health indicators (i.e. body mass index), and psychosocial factors related to healthy eating (self-efficacy, intentions, and knowledge) is important to further develop this concept. Only one study to date has examined weight status with safe food consumption patterns [14], and while there is some literature focusing on food safety behaviors and psychosocial factors (including self-efficacy and intentions), the majority of this in teen and young adult populations[15]–[18] and little information is known about these associations in adults, creating a need for additional exploration in this area.

Identification of health indicators related to risky food safety behaviors can be used to create programs and policies that are dually functioning to promote positive change on that health indicator (i.e. reductions in Body Mass Index (BMI), promotion of health-related psychosocial factors) and improve food safety behaviors. Well-designed, multi-faceted public health programs would have the potential to create greater impact and promote decreased health care spending associated with both foodborne illness and the associated health concerns.

This article reports on baseline data from a nutrition and physical activity intervention designed to develop and evaluate the effects of an intensive, church-based, diabetes prevention program targeting African Americans. We explore the relationship between food safety behaviors and multiple sociodemographic, health, and nutrition-related psychosocial factors and further investigate the following questions:

1. How frequently do African American churchgoers in Baltimore City perform recommended food safety practices?
2. What characteristics of African American churchgoers are related to food safety behaviors?
3. Are health indicators (BMI) and health-related psychosocial factors associated with food safety behaviors?

## Methods

### Sampling

Data were collected from June 2010 to August 2011 as part of baseline data collection for the Healthy Bodies, Healthy Souls (HBHS) study, a church-based diabetes intervention trial. The HBHS study included a convenience sample of 285 participants recruited from 9 different African American churches in Baltimore City located in low-income census tracks. The 9 churches that participated in HBHS were recruited in 3 waves with 3 churches in each wave. Baseline data collection occurred prior to the start of each wave, creating an extended timeframe for the baseline data collection period. Food safety behaviors were examined in this population in coordination with a pilot study examining the safety (microbial presence) of food and access to food, in the low-income, predominately African American neighborhoods surrounding the 9 HBHS churches.

All participating churches were located in low-income, inner-city neighborhoods, however, the churchgoers who attended these churches did not necessarily live in the neighborhood surrounding the church and were of mixed socioeconomic status (as seen in Table 1); thus, results are not necessarily representative of the church neighborhoods. The analyses presented in this paper focus on a sub-sample of 153 participants from whom we collected an additional food safety questionnaire. The sub-sample was not significantly different from the total study sample (at the p<0.05 level) in any of the variables of interest including demographic characteristics or health indicators.

**Table 1:** Descriptive statistics for the sample of Baltimore African American churchgoers (n = 153).

	Total Sample
Sex	
Female	79.9%
Male	20.1%
Education level	
Less than a college degree	56.9%
College degree	43.1%
Age, years (mean±SD)	46.01±13.80
BMI (mean ±SD)	32.13±7.01
Weight Status	
Normal weight (BMI 18.5–24.9)	12.93%
Overweight (BMI 25.0–29.9)	26.71%
Obese (BMI≥30.0)	60.36%
Annual household income (dollars)	
Less than 30,000	22.20%
30,001–50,000	20.30%
50,001–80,000	25.50%
More than 80,000	22.90%
Declined to answer	9.20%
Food security level	
Food secure	63.8%
Household food insecure	21.7%
Child/adult food insecure	14.5%
Material style of life score (mean±SD)	16.33±10.18

To be eligible to participate in this study, churchgoers had to be African American, over the age of 18, and attend one of the participating churches 2–3 times per month. Participants were excluded from the study if they were currently pregnant, if they self-reported having diabetes mellitus, and/or if they reported having a condition that prohibited them from doing moderate-intensity physical activity.

### Procedures for Data Collection

Permission was obtained from church leadership at all churches involved to recruit congregation members into the study program. After church services, data collectors performed initial eligibility screenings at the church, and eligible participants were scheduled for a data collection interview. Interviews occurred in the setting that was most convenient for the participant, including participant homes, church offices, and offices at the Johns Hopkins Bloomberg School of Public Health. The entire interview lasted 2 to 3 hours and included administration of a quantitative food frequency questionnaire [19] and the International Physical Activity Questionnaire [20], which were not included in this analysis. Trained data collectors took all anthropometric measures using standard procedures. Repeated measures of anthropometric data were taken and averaged to increase accuracy. If a participant refused measurement, self-reported height and weight measures were used. This situation occurred in 6.53% (n = 10) of the 153-person sub-sample. Participants were compensated for completion of the interview with a gift card. Written informed consent was obtained from all participants. The Johns Hopkins Bloomberg School of Public Health Institutional Review Board approved the study protocol and all data collection instruments.

### Description of Adult Impact Questionnaire

An 117-item Adult Impact Questionnaire (AIQ) was developed based on similar instruments used previously [21] and formative research conducted in the area. The AIQ was divided into sections. The relevant components of the AIQ include the following:

### Meal Preparation & Food Safety Behaviors and Concerns

Meal preparation questions included frequency of meal preparation for the individual and for the household. Food safety was assessed by 7 questions regarding food safety behaviors and overall concern with food safety. The questions were developed based on the behaviors identified in the Healthy People 2020 objectives [9] and evaluated according to published methods with appropriate adjustments [4].

### Psychosocial Factors

The psychosocial factors addressed were healthy eating self-efficacy, healthy eating intentions, and food knowledge. The self-efficacy section was designed to determine the participant’s confidence in his/her ability to make healthy choices related to dietary intake. Healthy eating intentions were determined by asking the participants to report which behavior he/she would “really choose” (as opposed to selecting the choice they thought was healthiest). The food knowledge section was designed to determine the level of food and nutrition knowledge of the individual and the ability of the individual to interpret a nutrition facts label. The psychosocial measures used here were taken from previous research studies conducted in this population [21].

### Demographics, Health History, and Anthropometrics

Sociodemographic information collected included marital status, education level, current employment, household income, and a Material Style of Life (MSL) scale, which is used as a proxy for socioeconomic status [22]. Anthropometric measurements included height, weight, waist circumference, and blood pressure. BMI was calculated from height and weight measurements by taking weight in kilograms divided by height in meters squared.

### Food Security

Food insecurity is defined as occurring whenever the availability of nutritionally adequate and safe foods, or the ability to acquire acceptable foods in socially acceptable ways, is limited or uncertain [23]. Food insecurity can occur on multiple levels. Based on the Cornell/Radimer instrument used in this study [24] food security is categorized as: food secure, household food insecure, individual (adult) food insecure, and individual (child) food insecure. Household food insecurity is present when household stores are depleted, decreasing quality and safety of available food and creating anxiety about food supplies. Individual level food insecurity occurs when there is inadequate energy intake or nutrient intake, feelings of deprivation or restricted choice of foods causing abnormal meal patterns [23]. Individual level food insecurity can occur in adults and/or children with the individual (child) level of food insecurity considered to be the most severe [23].

### Data Analysis

Data were analyzed using the IBM SPSS [25] statistical software version 19. The level of significance set for all analyses was p<0.05. For questions relating to food safety behaviors and psychosocial factors, scores and scales were established.

### Description of Scales and Scoring

#### Food safety behaviors score

Food safety behaviors and overall concern with food safety were assessed by a series of 7 questions. Six of the 7 questions asked about specific food safety behaviors (i.e. hand washing, separating raw meat from cooked foods, proper refrigerating and reheating procedures) similar to the behaviors targeted in the Healthy People 2020 objectives [9]. These questions were used to calculate an overall food safety score for each participant. The seventh question was excluded from this score because it collected information about the individual’s overall concern with food safety rather than a specific food safety behavior. Five of the 6 questions included in the food safety behaviors score asked about the frequency of practicing proper food safety behaviors, with responses of “never,” “sometimes,” “usually,” “always,” and “not applicable.” Responses indicating lower frequency of performing these behaviors indicated riskier food safety behaviors. The answers of “never” and “sometimes,” signifying less frequency, received negative scores of −2 and −1, respectively, while responses of “usually” and “always,” indicating higher frequency of the behavior, received positive scores of +1 and +2, respectively. A neutral response of “not applicable” was scored as zero. The six questions were summed to get an overall score with a possible range of −12, indicating very risky food safety behaviors, to +12, indicating the least risky food safety behaviors. The assignment of response values and scoring methods used are based upon similar methods recently published in the literature [4].

### Psychosocial Factors

The scoring for the psychosocial factors scales is based on methods used in previous studies in Baltimore using similar data collection instruments [21].

### Healthy Eating Self-efficacy Score

Based upon 13 questions, the healthy eating self-efficacy score determined the participants’ confidence level in their ability to make healthy eating decisions by asking participants to rate how difficult or easy it would be for them to do certain health-related behaviors on a regular basis. For example, participants were asked how difficult it would be for them to choose diet soda rather than regular soda. Responses of “very easy,” “somewhat difficult,” “very difficult,” or “impossible” were based on a 4-point likert scale from 4 to 1, with higher scores indicating increased self-efficacy. Possible scores ranged from 13 to 52. The sample mean was fairly high at 45.70 (SD = 4.35). The scale has moderate internal validity with a Cronbach’s alpha of .693.

### Healthy Eating Intentions Score

Healthy eating intentions were measured by 14 questions eliciting the participants’ intentions for selecting more or less healthy choices when making health-related behavior decisions. To obtain more accurate responses, participants were encouraged to report what they actually intended to do the next time they were given the hypothetical choice, rather than selecting an answer based on how healthy or unhealthy they thought the response was. For example, participants were asked, “If you wanted a snack, what would you pick?” with responses of “potato chips,” “fruit,” or “reduced-fat baked chips.” For these questions the healthiest option received 3 points, the intermediate option received 2 points, and the least healthy option received 1 point. The scale has a minimum possible score of 14, a maximum possible score of 42, and a Cronbach’s alpha of.687. The mean healthy eating intentions score of the sample was 27.02 (SD = 4.97).

### Food Knowledge Score

The food knowledge score was calculated from responses to 18 questions about general knowledge of foods, including a component where participants were given a sample food label and asked to answer questions based on the label. All responses were multiple choice, except for the nutrition label questions, which required a numeric or yes/no response. The food knowledge score is calculated by summing the number of correct responses. The minimum possible score for this scale is 0 with a maximum possible score of 18. In this sample, scores had a mean of 12.44 (SD = 3.01, Cronbach’s alpha of .650).

### Statistical Tests

Descriptive statistics were run on all variables of interest, including participant demographics and the individual and scored responses to the food safety behaviors questions. Simple linear regressions were used to determine the relationships of food safety behaviors with demographic, psychosocial, and health-related variables. The simple linear regression analyses (not shown) along with a priori hypotheses of related variables based on the literature [4], [10]–[18] were used to inform the development of the multiple linear regression models. A multiple linear regression analysis was conducted to examine the relationship between food safety behaviors scores and demographic characteristics, self-efficacy related to healthy eating, and BMI. Other multiple linear regression analyses were conducted to determine factors (including food safety behaviors and health indicators) associated with psychosocial variables (self-efficacy for healthy eating, healthy eating intentions, and food knowledge). Multiple regression models controlled for demographic variables such as education level, gender, location of residence (Baltimore City resident versus non-resident) and income. Cross-sectional data are used in these analyses, so causality of the relationships is unknown and could benefit greatly from further examination. A significance level of p<0.05 was set for all regression analyses.

## Results

### General Description of the Study Sample

The sample was predominately female (79.9%), middle-aged (46.01±13.80) with slightly less than half (43.1%) of the sample reporting having a college degree (associates, bachelors, or higher) (Table 1). The annual household income of the study sample varied widely with approximately 22% having household incomes less than $30,000 per year and nearly 23% above $80,000 per year. Based upon Cornell/Radimer food security questionnaire, 36.2% of households were classified as having some level of food insecurity (household, individual (adult), and/or individual (child) food insecurity). The mean BMI of the sample was in the obese category (32.13±7.01), with only 12.93% of the sample classified as normal weight (BMI 18.5–24.9). High rates of overweight and obesity occurred in the sample with 26.71% classified as overweight (BMI 25.0–29.9), and 60.36% classified as obese (BMI of 30 or higher). The high overweight and obesity rates seen in this sample are not surprising given the high overall rates of overweight and obesity in African Americans in Baltimore City (74%) [26], and rates among African American women over 40 years old nation-wide (over 80%) [27].

### Frequency of Food Safety Behaviors Among the Study Sample

Table 2 provides descriptive statistics of the food safety questions asked in this study. Participants reported a high overall concern about food safety. The mean food safety behaviors score of the sample was 5.26 (SD = 4.01), indicating that African American churchgoers in Baltimore had moderately safe food handling practices. There was inconsistency in the implementation of the food safety behaviors as evidenced by mean reported frequency of certain behaviors being much higher than others. Hand washing and cleaning cooking tools before and after contact with raw meat, poultry, and seafood and separation of raw meat and poultry from other foods were performed with the highest frequency. Usual methods for thawing meat, fish, or poultry was the lowest scoring food safety behavior, with more than half of the participants’ responses to this question considered to be unsafe methods, causing negative scores. Scores for questions addressing the proper refrigeration and reheating of leftovers were also lower, with slightly more than 1/3 of the sample reporting the lowest frequency responses of “sometimes” or “never.”

**Table 2:** Descriptive statistics for food safety behavior questions.

	(Mean±SD)^a^
1. How often do you wash hands, utensils, and cutting boards before and after contact with raw meat, poultry, seafood, and eggswith hot/warm soap water?	1.80±0.68
2. How often do you keep raw meat and poultry apart from the foods that won’t be cooked in your fridge or grocery cart?	1.28±1.26
3. How often do you stir, rotate the dish, and cover food when microwaving?	0.88±1.43
4. How often do you bring sauces, soups and gravies to a rolling boil when reheating?	0.33±1.56
5. How often do you refrigerate or freeze leftovers and take-out foods within 2 hours?	0.60±1.47
6. How do you usually thaw frozen meat, fish or poultry?	0.01±1.71
**Food safety behavior score** b	**5.26**±**4.01**
7. How concerned are you about food safety? c	0.80±0.49

a Questions 1–5 are scored on a 5-point likert scale with possible responses (and associated value) of Never (−2), Sometimes (−1), N/A (0), Usually (1), and Always (2). Question 6 had possible responses of: On the counter (−2), In the Sink (−2), In water (−1), N/A (0), In the microwave (1), and In the refrigerator (2). Lower scores indicate riskier food safety behaviors.

b Food safety behavior score was created by summing questions 1–6. Total range of possible food safety behavior scores: −12 to +12. Question 7 was excluded in calculation of the food safety behavior score because it does not refer to a behavior.

c Based of a scale of −1 to +1, with a responses of not at all concerned (−1), somewhat concerned (0), very concerned (1).

### Relationships with Food Safety Behaviors Scores

Multiple linear regression analyses, controlling for demographic variables (including age, sex, location of residence, education level, income, and number of meals prepared per week), were conducted to investigate relationships between health-indicators, psychosocial factors, and food safety behaviors. Strong positive relationships were identified between healthy eating self-efficacy scores and food safety behaviors scores outcomes (β [95% CI] = 0.213 [0.062, 0.364], p = 0.006), and strong inverse relationships were seen between BMI and food safety behaviors scores (β [95% CI] = −0.141[−0.237, −0.044], p = 0.004) in this multivariate analysis (Table 3). On the food safety behaviors scale with a possible range of −12 to 12, there was a 0.141 point decrease in food safety behaviors score for each 1 unit increase in BMI, controlling for age, gender, location of residence, income, number of meals prepared, and self-efficacy. There was a 0.213 point increase in food safety behaviors score for each 1 unit increase in self-efficacy score, controlling for age, gender, location of residence, number of meals prepared and BMI.

**Table 3:** Regression analysis examining factors associated with food safety behavior scores as an outcome in a sample of Baltimore African American churchgoers.

Variable	β (95%CI)	p-value
N	133	
R^2^	0.158	
Age (years)	**−**0.012 (**−**0.062, 0.037)	0.623
Sex (male = 1 vs. female = 2)	0.682 (**−**1.013, 2.378)	0.427
Location of Residence (other = 0 vs. Baltimore City resident = 1)	0.207 (**−**1.183, 1.597)	0.769
Education Level (< College Degree = 0 vs. College Degree = 1)	1.064 (**−**0.299, 2.426)	0.125
Annual Household Income^a^	0.016 (**−**0.011, 0.042)	0.255
Number of Meals Prepared per Week	0.032 (**−**0.037, 0.101)	0.356
Healthy Eating Self-Efficacy Score	0.213 (0.062, 0.364)	0.006*
BMI	**−**0.141 (**−**0.237, **−**0.044)	0.004*

* p-value<0.05.

β represents an unstandardized beta coefficient.

R^2^ represents the variance in the outcome that is explained by the variables in the model.

All variables adjusted for in the model are included in the table above.

a Annual Household Income categorized as 1 = $0–30,000 2 = $30,001–50,000 3 = $50,001–80,000 4 =  over $80,000.

Associations between food safety behaviors and demographic variables included in this regression model (including gender, education, and others) did not reach statistically significant levels (p<0.05), and therefore claims about these relationships can not be made.

### Associations with Food-related Psychosocial Factors

Table 4 examines the relationships of psychosocial variables with food safety behaviors, when controlling for BMI and sociodemographic characteristics. Scores for self-efficacy and food knowledge were not normally distributed and were transformed for these analyses (food knowledge scores were squared and self-efficacy scores were cubed); due to the transformations, standardized betas are reported. In these multiple linear regression analyses, food safety behaviors scores had significant positive relationships with healthy eating self-efficacy (Std. β = 0.250, p = 0.005) and intentions (Std. β = 0.178, p = 0.041), and they had a positive relationship with food knowledge scores that did not reach statistical significance. Increased age was associated with higher healthy eating self-efficacy scores (Std. β = 0.174, p = 0.045) and having a college degree was associated with increased food knowledge scores (Std. β = 0.344, p = 0.000) and healthy eating intentions scores (Std. β = 0.205, p = 0.020). Interestingly, higher BMI scores were positively associated with higher food knowledge scores (Std. β = .235, p = .004). The models relating food safety scores, demographics, and BMI to psychosocial factors account for moderate amounts of the variability (11.8%−22.3%) seen in the food-related psychosocial variables.

**Table 4:** Regression analyses examining variables associated with food-related psychosocial factors as outcomes among African American churchgoers.

Variable	Food knowledge score^a^			Healthy eating self-efficacy score^b^		Healthy eating intentions		
N	134			133		134		
R^2^	0.223			0.125		0.118		
		Std. β	p-value	Std. β	p-value	Std. β	p-value	
Food safety behavior score		0.086	0.296	0.250	0.005*	0.178	0.041*	
Age (years)		**−**0.126	0.124	0.174	0.045*	0.127	0.144	
Sex (male = 1 vs. female = 2)		0.049	0.538	0.082	0.335	0.081	0.342	
Education level (< College Degree = 0 vs. College Degree = 1)		0.344	0.000*	**−**0.052	0.553	0.205	0.020*	
Annual household income^c^		**−**0.118	0.139	0.076	0.373	0.118	0.167	
BMI		0.235	0.004*	0.112	0.199	**−**0.027	0.748	

Std. β indicates standardized beta, which was used because of the transformed variables.

R^2^ is the variance in the outcome that is explained by the variables in the model.

All variables adjusted for in the model are included in the table above.

a Square transformed to approximate a normal distribution.

b Cube transformed to approximate a normal distribution.

c nnual Household Income categorized as 1 = $0–30,000 2 = $30,001–50,000 3 = $50,001–80,000 4 =  over $80,000.

* p-value<0.0.

## Discussion

In this sample of middle-income African American churchgoers, food safety behaviors are being practiced with moderate regularity, but there is still a clear need for reduction of risky food handling practices. Hand washing and avoidance of cross contamination were reported more frequently. Proper thawing, refrigeration of leftovers, reheating and microwaving were reported less frequently.

In this analysis we found that, after adjusting for education and other demographic variables, individuals with higher BMIs were more likely to perform risky food safety behaviors, and individuals with higher healthy eating self-efficacy and higher healthy eating intentions were less likely to perform risky food safety behaviors. These findings are interesting and novel because much effort has gone into determining the association between demographic factors (gender, age, education level, income, race/ethnicity, metropolitan status, and geographic location) and risky food safety behaviors[4], [7], [10]–[14], yet BMI and health-related psychosocial factors are rarely included among the characteristics examined in adult populations. We are aware of only one study [14] to date that has examined these relationships by weight status. This study found that individuals with a BMI over 25 were more likely than their normal weight counterparts to consume undercooked ground beef [14], indicating tendencies toward risky food consumption among overweight individuals in this one area. A majority of studies that examine associations between self-efficacy or other psychosocial variables and food safety behaviors are in youth and young adult populations [15]–[18], with little known about these relationships among adults. The results of the studies in young adults are consistent with our findings that increased self-efficacy and intentions are related to better food safety habits.

One interesting and important consideration in examining the relationship between food safety behaviors, and psychosocial factors related to healthy eating, and BMI is causality. This study is limited in that cross-sectional data only allows us to speculate on the causal relationship of these factors. Determining the directionality of the relationships is necessary to guide the development of strategies appropriate for addressing these health risks. Expanded research is needed to further clarify these relationships and examine the public health implications associated with them.

We hypothesize that one potential explanation for the association between food safety behaviors and BMI found in this study is that individuals with lower BMI scores are more likely to have an increased focus on healthy behaviors overall, including increased attention paid to nutrition and food preparation, which assists them in maintaining a lower BMI. The association of higher healthy eating self-efficacy and intentions scores with food safety behaviors lends evidence to this hypothesis, showing that participants who had increased confidence in their abilities to select healthier choices and greater intentions to consume a healthy diet reported more frequent use of proper food safety behaviors. These relationships are of interest from both the food safety and weight management perspectives, and further investigations are warranted.

A limitation of this study was that the participants were volunteers, and they potentially differed from members of the same churches who did not participate. The study sample also had a very high percentage of individuals who were overweight or obese, which must be considered when interpreting the study findings. However, the overweight and obesity rates in this sample are similar to what is found in African American populations in Baltimore City [26], and to African American women across the U.S. who are over the age of 40 [27], and therefore the sample is likely to be representative of this population. Additionally, because data for this study was collected primarily for the HBHS study, some vulnerable populations were excluded based on the HBHS eligibility criteria (pregnant women and people with diabetes). Future analyses should include these vulnerable populations as they are at higher risk of negative effects if they are exposed to foodborne illnesses. Additional studies are needed in more diverse samples to confirm the external validity of the results.

We must also consider that the food safety behavior data collected in this study was self-reported. In the literature it has been found that individuals often over-report the practice of food safety behaviors [6], [7], indicating that there is the potential for an even greater need for improvements in food safety behaviors than what is reported here. Finally, this study focused on individual factors that are related to food safety behaviors, and was unable to measure and report on environmental factors that may also have a significant impact on the food safety behaviors. Future studies would benefit from taking into account both, individual and environmental factors when examining these relationships.

Efforts toward helping Americans reach the Healthy People 2020 goal of increasing individuals’ food safety knowledge and promoting proper food safety behaviors must be continued. The results of this study provide additional insight on how to best achieve this goal. Differences in frequency of conducting food safety behaviors were found in this study, which is consistent with the literature [5]. Targeting food safety messages to populations with riskier food safety behaviors has been shown to be effective [28]. The relationships between BMI and healthy eating self-efficacy and intentions, and food safety behaviors found here are new. Individuals with higher BMIs are a potential group for which targeted messages may be helpful, but have not yet been conducted. Programs promoting proper food handling procedures coupled with methods to increase self-efficacy and intentions for consuming healthy, safe foods could be an appropriate means for addressing this dual issue. Weight management programs that require participants to prepare their own food are another potential venue for these messages. Weight management programs should be aware of the potential for improper food handling procedures among participants and should promote food safety messages along with their other food preparation and dietary recommendations.

The results of this study indicate that additional research is needed to explore the relationships between food safety behaviors and weight status and other psychosocial variables, particularly self-efficacy and intentions for healthy eating. Future studies should include larger, more diverse samples and increased measures of food safety, environmental factors, and other health-related variables.

## References

[1] Scallan E (2011) Foodborne illness acquired in the United States–Major pathogens. Emerg Infect Dis 17: 7–16. 21192848 10.3201/eid1701.P11101 PMC3375761

[2] Hoffman S (2011) U.S. food safety policy enters a new era. Amber Waves: The economics of food, farming, natural resources, and rural America. Available: http://www.ers.usda.gov/AmberWaves/December11/Features/FoodSafetyPolicy.htm. Accessed: 2012 January 29.

[3] Busby J, Roberts T, Lin C, MacDonald J (1996) Bacterial foodborne diseases: Medical costs and productivity losses. Food and Consumer Economics Division, Economic Research Service, U.S. Department of Agriculture Report No. 741. Available: http://www.ers.usda.gov/publications/aer741/AER741fm.PDF. Accessed: 2012 January 29.

[4] Fein SB, Lando AM, Levy AS, Teisl MF, Noblet C (2011) Trends in U.S. consumers’ safe handling and consumption of food and their risk perceptions, 1988 through 2010. J Food Prot 74: 1513–1523. 21902921 10.4315/0362-028X.JFP-11-017

[5] Lando A, Verrill L (2006) 2006 FDA/FSIS food safety survey topline frequency report. Available: http://www.fda.gov/Food/ScienceResearch/ResearchAreas/ConsumerResearch/ucm080374.htm. Accessed 2012 February 7.

[6] Anderson JB, Shuster T, Hansen KE, Levy AS, Volk A (2004) A camera’s view of consumer food-handling behaviors. J Am Diet Assoc 104: 186–191 doi:10.1016/j.jada.2003.11.010. 14760565 10.1016/j.jada.2003.11.010

[7] Redmond EC, Griffith CJ (2003) Consumer food handling in the home: a review of food safety studies. J Food Prot 66: 130–161. 12540194 10.4315/0362-028x-66.1.130

[8] U.S. Department of Agriculture and U.S. Department of Health and Human Services. Dietary Guidelines for Americans, 2010. 7th Edition. (2010). Washington DC. Available: http://www.health.gov/dietaryguidelines/. Accessed: 2012 January 29. 10.3945/an.111.000430 PMC3090168 22332062

[9] U.S. Department of Health and Human Services. Office of Disease Prevention and Health Promotion. Healthy People 2020 (2010). Washington, DC. Available: http://healthypeople.gov/2020/topicsobjectives2020/overview.aspx?topicid=14. Accessed: 2012 February 3.

[10] Patil SR, Cates S, Morales R (2005) Consumer food safety knowledge, practices, and demographic differences: findings from a meta-analysis. J Food Prot 68: 1884–1894. 16161688 10.4315/0362-028x-68.9.1884

[11] Altekruse SF, Yang S, Timbo BB, Angulo FJ (1999) A multi-state survey of consumer food-handling and food-consumption practices. Am J Prev Med 16: 216–221. 10198661 10.1016/s0749-3797(98)00099-3

[12] Samuel M, Vuglia D, Koehler K, Marcus D, Deneen V, et al (2007) Consumption of risky food among adults at high risk for severe foodborne diseases: Room for improved targeted prevention messages. J Food Saf 27: 219–232.

[13] Roseman M, Kurzynske J (2006) Food safety perceptions and behaviors of Kentucky consumers. J Food Prot 69: 1412–1421. 16786865 10.4315/0362-028x-69.6.1412

[14] Zhang P, Penner K, Johnston J (1999) Prevalence of selected unsafe food-consumption practices and their associated factors in Kansas. J Food Saf 19: 289–297.

[15] Byrd-Bredbenner C, Abbot JM, Wheatley V, Schaffner D, Bruhn C, et al (2008) Risky eating behaviors of young adults-implications for food safety education. J Am Diet Assoc 108: 549–52. 18313439 10.1016/j.jada.2007.12.013

[16] Abbot JM, Byrd-Bredbenner C, Schaffner D, Bruhn CM, Blalock L (2009) Comparison of food safety cognitions and self-reported food-handling behaviors with observed food safety behaviors of young adults. Eur J Clin Nutr 63: 572–9. 18000516 10.1038/sj.ejcn.1602961

[17] Haapala I, Probart C (2004) Food safety knowledge, perceptions, and behaviors among middle school students. J Nutr Educ Behav 36: 71–76. 15068755 10.1016/s1499-4046(06)60136-x

[18] Shapiro MA, Porticella N, Jiang LC, Gravani RB (2011) Predicting intentions to adopt safe home food handling practices. Applying the theory of planned behavior. Appetite 56: 96–103. 21115082 10.1016/j.appet.2010.11.148

[19] Sharma S, Cao X, Arcan C, Mattingly M, Jennings S, et al (2009) Assessment of dietary intake in an inner-city African American population and development of a quantitative food frequency questionnaire to highlight foods and nutrients for a nutritional invention. Int J Food Sci Nutr. 60 Suppl 5155–167. 10.1080/09637480902755061 19353421

[20] Craig CL, Marshall AL, Sjöström M, Bauman AE, Booth ML, et al (2003) International physical activity questionnaire: 12-country reliability and validity. Med Sci Sports Exerc 35: 1381–1395. 12900694 10.1249/01.MSS.0000078924.61453.FB

[21] Suratkar S, Gittelsohn J, Song H-J, Anliker JA, Sharma S, et al (2010) Food insecurity is associated with food-related psychosocial factors and behaviors among low-income African American adults in Baltimore City. J Hunger Environ Nutr 5: 100–119 doi: 10.1080/19320240903582661.

[22] Erber E, Beck L, Hopping BN, Sheehy T, De Roose E, et al (2010) Food patterns and socioeconomic indicators of food consumption amongst Inuvialuit in the Canadian Arctic. J Hum Nutr Diet 23 Suppl 159–66 doi: 10.1111/j.1365–277X.2010.01097.x. 21158963 10.1111/j.1365-277X.2010.01097.x

[23] Radimer KL (2002) Measurement of household food security in the USA and other industrialised countries. Public Health Nutr 5: 859–864. 12633509 10.1079/PHN2002385

[24] Kendall A, Olson CM, Frongillo EA (1995) Validation of the Radimer/Cornell measures of hunger and food insecurity. J Nutr 125: 2793–2801. 7472659 10.1093/jn/125.11.2793

[25] Statistical Package for the Social Sciences, Version 19 (2010). IBM Corporation. Chicago, IL.

[26] Maryland Behavioral Risk Factor Surveillance System (2010) BRFSS – Results 2012. Available: http://www.marylandbrfss.org/cgi-bin/broker.exe. Accessed 2012 August 10.

[27] Wang Y, Beydoun MA (2007) The obesity epidemic in the United States–gender, age, socioeconomic, racial/ethnic, and geographic characteristics: A systematic review and meta-regression analysis. Epidemiol Rev 29: 6–28. 17510091 10.1093/epirev/mxm007

[28] Jacob C, Mathiasen L, Powell D (2010) Designing effective messages for microbial food safety hazards. Food Control 21: 1–6.

