# Correction: Risky Food Safety Behaviors Are Associated with Higher BMI and Lower Healthy Eating Self-Efficacy and Intentions among African American Churchgoers in Baltimore

**DOI:** 10.1371/annotation/62acf365-13e3-457c-a757-dd61c85214d8

**Published:** 2013-05-31

**Authors:** Elizabeth Anderson Steeves, Ellen Silbergeld, Amber Summers, Lenis Chen, Joel Gittelsohn

The correct version of the title is: Risky Food Safety Behaviors Are Associated with Higher BMI and Lower Healthy Eating Self-Efficacy and Intentions among African American Churchgoers in Baltimore

The correct citation is: Anderson Steeves E, Silbergeld E, Summers A, Chen L, Gittelsohn J (2012) Risky Food Safety Behaviors Are Associated with Higher BMI and Lower Healthy Eating Self-Efficacy and Intentions among African American Churchgoers in Baltimore. PLoS ONE 7(12): e52122. doi:10.1371/journal.pone.0052122 

